# Books Reviewed

**Published:** 1866-07

**Authors:** 


### Books Reviewed.

Transactions of the Medical Society of the State of New York for the year 1865.

The present volume of the Transactions of the State Medical Society is one of the most interesting of its publications. The articles published, furnish us with additional evidence of the increasing interest of the profession in the prosperity of the State Medical Society, as well as in the advancement of medical science; many of them contain suggestions of practical value, some of which are, perhaps, new to many of the profession.—Among the papers of more especial interest we notice that by Cyrus Ramsay, M. D., LL.D., on "Small-pox, or remarks on Vaccination; the possibility of communicating Syphilis and other diseases through the Vaccine Virus." In tracing the history of vaccination, the writer furnishes us with some interesting statistics regarding the comparative mortality rate from small-pox, prior and subsequent to the introduction of it. It appears that the deaths from this disease in London from the year 1661 to 1684, both inclusive, comprising a period of 24 years, amounted to

28,479, while out of 2,550 cases occurring from the year 1826 to 1844, both inclusive, in the London Small-pox Hospital, 164 or 7 per cent. died. The mortality arising from this disease, in the city of New York, from 1854 to 1864, a period of ten years, was 3,618; the deaths during the same period in Philadelphia, being 1,984, in Boston 206, and in Baltimore 485. He considers the total extermination of small-pox an impossibility, but should vaccination be practiced every seven or ten years, the disease would be so largely diminished, and those that did occur would be of so mild a character as to require but little attention. From the table of re-vaccinations prepared by Dr. Heine, out of 44,009 cases, 20,000 took the disease perfectly, 9,009 imperfectly, and 15,000 not at all.

"The report on the condition of the insane poor in the county poor-houses of New York," by Sylvester D. Willard, of Albany, to the Legislature of the State, represent a condition of wretchedness so appalling in most of these institutions, as to demand immediate attention on part of the different counties. Dr. Willard has made his investigations in the summer, "when the suffering from want of care and clothing is less than in the winter, and consequently it does not show the state of things as bad as they really exist at some seasons of the year. Cleanliness and ablutions are not enforced; indeed very few of the institutions have even the conveniences for bathing, and many of the buildings are inadequately supplied with water. In a few instances the insane are not washed at all, and their persons besmeared with their own excrements are unapproachingly filthy, disgusting and repulsive. In some violent cases the clothing is torn and strewn about the apartments, and the lunatics continue to exist in wretched nakedness, having no clothing, and sleeping upon straw, wet and filthy with excrements, and unchanged for several days. There exists gross inattention to ventilation, and in frequent instances these unfortunates are denied even the fresh air of heaven. The buildings in many instances are but miserable tenements, and were erected without any regard to ventilation. In some of these buildings the insane are kept in cages and cells, dark and prison-like, as if they were convicts, instead of the life-weary, deprived of reason. They sleep on straw like animals, and there are scores who endure the piercing cold and frost of winter without either shoes or stockings being provided for them." Such is the lamentable condition of institutions called *charitable* and *philanthropic.*

We should be happy to notice some other essays, would space permit our doing so. In conclusion we wish an increased prosperity to an association that embodies amongst its members the *elite* of the medical profession of the State of New York. C. N.

